# Changes in corticospinal and spinal reflex excitability through functional electrical stimulation with and without observation and imagination of walking

**DOI:** 10.3389/fnhum.2022.994138

**Published:** 2022-09-26

**Authors:** Naotsugu Kaneko, Atsushi Sasaki, Hikaru Yokoyama, Yohei Masugi, Kimitaka Nakazawa

**Affiliations:** ^1^Department of Life Sciences, Graduate School of Arts and Sciences, The University of Tokyo, Tokyo, Japan; ^2^Department of Mechanical Science and Bioengineering, Graduate School of Engineering Science, Osaka University, Osaka, Japan; ^3^Japan Society for the Promotion of Science, Tokyo, Japan; ^4^Institute of Engineering, Tokyo University of Agriculture and Technology, Tokyo, Japan; ^5^School of Health Sciences, Tokyo International University, Saitama, Japan

**Keywords:** action observation, motor imagery, functional electrical stimulation, motor evoked potential, Hoffmann-reflex

## Abstract

Functional electrical stimulation (FES), a method for inducing muscle contraction, has been successfully used in gait rehabilitation for patients with deficits after neurological disorders and several clinical studies have found that it can improve gait function after stroke and spinal cord injury. However, FES gait training is not suitable for patients with walking difficulty, such as those with severe motor paralysis of the lower limbs. We have previously shown that action observation combined with motor imagery (AO + MI) of walking induces walking-related cortical activity. Therefore, we combined FES, which alternately generates dorsiflexion and plantar flexion, with AO + MI as an alternative to gait training. The present study investigates the transient effects of 20-min of FES simultaneously with and without AO + MI of walking on corticospinal and spinal reflex excitability in able-bodied participants. We measured motor evoked potentials and Hoffmann-reflexes to assess corticospinal and spinal reflex excitability at rest before and after the 20-min FES with and without the AO + MI. Our results show that FES without AO + MI did not change excitability (*p* > 0.05), while FES with AO + MI facilitated corticospinal excitability (*p* < 0.05). This facilitation likely occurred due to the synchronization of sensory inputs from FES and cortical activity during AO + MI. Facilitation was observed only in the dorsiflexor but not the plantar flexor muscle (*p* < 0.05), suggesting muscle specificity of the facilitation. These results demonstrate the effectiveness of combining FES with AO + MI and pave the way for novel neurorehabilitation strategies for patients with neurological gait deficits.

## Introduction

Functional electrical stimulation (FES) is a method for inducing muscle contraction to assist or restore motor function (Rushton, 2003; Carson and Buick, 2021). FES has been used in gait rehabilitation for patients with gait deficits after neurological disorders. Several clinical studies have reported that rehabilitation combined with FES gait training was more effective in improving gait function after stroke and spinal cord injury than gait training without FES (Burridge et al., 1997; Kottink et al., 2004; Daly et al., 2006; Robbins et al., 2006; Nightingale et al., 2007; Dickstein, 2008; Kapadia et al., 2014). In gait rehabilitation after stroke, FES is typically delivered to the ankle dorsiflexor muscles to prevent foot drop during the swing phase (Kottink et al., 2004; Robbins et al., 2006; Kesar et al., 2010), and to the ankle plantar flexor muscles to support force generation during the stance phase (Kesar et al., 2009; Hakansson et al., 2011). In post-stroke patients, FES gait training can increase walking speed and mitigate ankle and knee joint dysfunction (Kottink et al., 2004; Robbins et al., 2006; Kesar et al., 2009). Such improvements may be caused by neuroplastic changes or by the enhancement of muscle strength function.

Movement-related cortical activity and sensory inputs, and their synchronization play important roles in neuroplastic changes (Stefan, 2000; Wolters et al., 2003; Pascual-Leone et al., 2005). Previous studies have reported that cortical activity during walking depends on the walking phase (Wagner et al., 2012; Yokoyama et al., 2020). We hypothesize, that phase-dependent cortical activity should enhance the effects of FES; that is, in FES gait training, FES-induced sensory inputs in specific phases would interact with walking-induced cortical activity to induce neuroplastic changes. This is supported by the observation that in able-bodied participants corticospinal excitability is increased after FES gait training according to walking phases, but not after gait training alone (Kido Thompson and Stein, 2004). A previous study suggested that the corticospinal tract partially mediated the recovery of gait function through training after incomplete spinal cord injury (Thomas and Gorassini, 2005). Thus, synchronization of cortical activation with sensory inputs and the facilitation of corticospinal excitability are related to improvements of gait functions.

However, FES gait training is not suitable for patients with walking difficulty, such as those with severe motor paralysis of the lower limbs. Action observation (AO) and motor imagery (MI) are alternative methods for inducing walking-related cortical activity, without engaging in overt movement (Miyai et al., 2001; Iseki et al., 2008; Cevallos et al., 2015). AO can be defined as “the perception of other’s action” (Fadiga et al., 1995; Rizzolatti et al., 2001), while MI can be defined as “mental simulation or rehearsal of a movement without any motor output” (Decety, 1996). Both AO and MI of walking activate the premotor cortex and the supplementary motor area involved in actual walking (Miyai et al., 2001; Iseki et al., 2008; Kaneko et al., 2021). Furthermore, AO combined with MI (AO + MI) of walking partially induces phase-dependent activation of the sensorimotor cortex during walking (Yokoyama et al., 2020, 2021; Kaneko et al., 2021). Thus, we can expect that FES combined with AO + MI of walking in concurrent would induce similar neuroplastic changes that would be expected from synchronization of cortical activation and sensory inputs through FES gait training, e.g., the facilitation of corticospinal excitability (Kido Thompson and Stein, 2004).

Previous studies have shown that peripheral nerve stimulation (PNS) combined with AO + MI of hand movement and ankle dorsiflexion induced transient changes in cortical and spinal activity (Takahashi et al., 2019; Yasui et al., 2019). However, these studies used AO + MI and PNS for single-joint movements controlled by a single muscle, while to the best of our knowledge, no studies have targeted whole-body movements controlled by two muscles or more, such as walking. Therefore, the present study focused on AO + MI of walking combined with FES of ankle dorsiflexion and plantar flexion (i.e., PNS of the common peroneal and tibial nerves). Our recent study found no modulation of corticospinal and spinal motor neuron excitability after a 20-min AO + MI of walking alone (Kaneko et al., 2022), indicating that AO + MI by itself exerted minor effects on neural activity; however, we implied that combining it with additional treatment may induce transient changes in electrophysiological measures of corticospinal and spinal excitability. The purpose of the present study is to apply FES for dorsiflexion and plantar flexion simultaneously with and without AO + MI of walking and to investigate its transient effect on corticospinal and spinal reflex excitability, which is related to gait functions. We used motor evoked potential (MEP) and Hoffmann-reflex (H-reflex) measurement to assess corticospinal and spinal reflex excitability at rest before and after FES with and without the AO + MI. We hypothesize that FES with AO + MI in concurrent, which involves the synchronization of cortical activity and sensory inputs, induces greater transient changes in excitability than does FES without AO + MI.

## Materials and methods

### Participants

Ten healthy individuals with no history of neurological disorders participated in the present study [eight males and two females, age: 27.1 ± 2.7 years (24–34 years), height: 170.5 ± 8.1 cm (153–182 cm), and weight: 62.9 ± 6.7 kg (48–71 kg); mean ± standard deviation (SD), range in parentheses]. All participants provided written informed consent to participate in the study, and the experimental procedures were approved by the local ethics committee of the University of Tokyo. The study was performed in accordance with the Declaration of Helsinki (1964).

### Preparation for the functional electrical stimulation

Before the experiment, we synchronized FES timing (i.e., electrical stimulation of the common peroneal and tibial nerves) with the electromyographic (EMG) activity of the walker in the video. The details are presented in the following sections.

#### Video recording of walking and electromyographic activity

**FIGURE 1:**
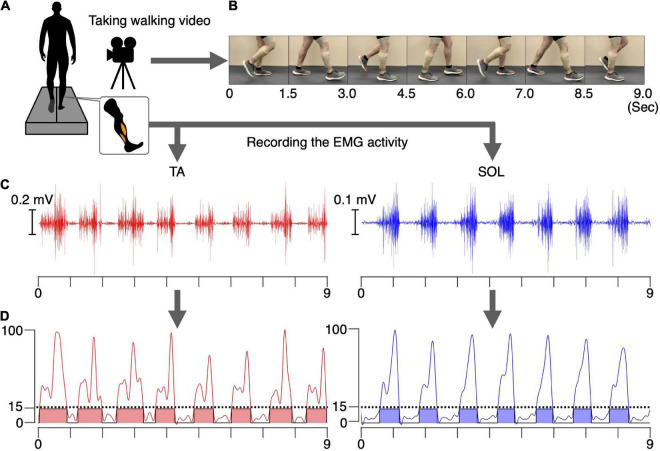
Preparation for the intervention. A video of a healthy male walking. The EMG signals in the TA and SOL muscles were synchronously recorded for 20 s **(A)**. Examples of 9 s of walking video **(B)**, raw EMG signals **(C)**, and the processed and normalized EMG signals in the TA and SOL muscles **(D)** are shown. The phases where the normalized EMG was above 15% were defined as the ON phases (colored areas), and phases where normalized EMG was below 15% were defined as the OFF phases.

We recorded a video of a healthy male (age: 26 years, height: 180 cm, weight: 80 kg) walking for 2 mins at 1.0 m/s on a treadmill (Bertec, Columbus, OH, United States) (Figure 1A). We synchronously recorded the EMG activity of the tibialis anterior (TA) and soleus (SOL) muscles using a wireless EMG system (Trigno Wireless System; DELSYS, Boston, MA, United States) (Figures 1B,C). Synchronization of the video and EMG recordings was achieved using a trigger box that simultaneously turns on a light and generates a reference signal. The EMG signals were filtered using a band-pass filter between 20 and 450 kHz. The analog signals were digitized at a sampling rate of 4 kHz using an analog-to-digital converter (Powerlab/16SP, AD Instruments, Castle Hill, NSW, Australia). The experimental procedure used 20 s of the walking video and the synchronized EMG signals. The walker did not participate in the experiment.

#### Determination of the on and off phases of electromyographic corresponding to the walking video

The recorded EMG signals in the TA and SOL muscles were detrended, high-pass filtered (zero-lag fourth-order Butterworth at 15 Hz), full-wave rectified, and smoothed with a low-pass filter (zero-lag fourth-order Butterworth at 5 Hz cutoff). The amplitudes of the processed EMG signals for each muscle were normalized to the maximum value of that muscle during the 20 s walking period (Figure 1D). Intervals during which the normalized EMG exceeded 15% were defined as ON, and intervals where the EMG signal was below 15% were defined as OFF phases for each muscle. During the intervention, in the TA muscle ON phase, the common peroneal nerve was stimulated, whereas in the SOL muscle ON phase the tibial nerve was stimulated. During the OFF phase, no PNS was performed. For both muscle, 17 separate ON phases were detected during total the 20 s duration. Average durations of the ON phase for the TA and SOL muscles were 636 ms and 550 ms, respectively. For common peroneal nerve stimulation, corresponding to the TA muscle, the total duration of the ON and OFF phases was 10.8 s and 9.2 s, respectively. For tibial nerve stimulation, corresponding to the SOL muscle, the total duration of the ON and OFF phases was 9.4 s and 10.6 s, respectively. The synchronized stimulus (see section “Video recording of walking and electromyographic activity”) was used for the intervention, as described in section “Experimental procedure.”

### Study design

**FIGURE 2:**
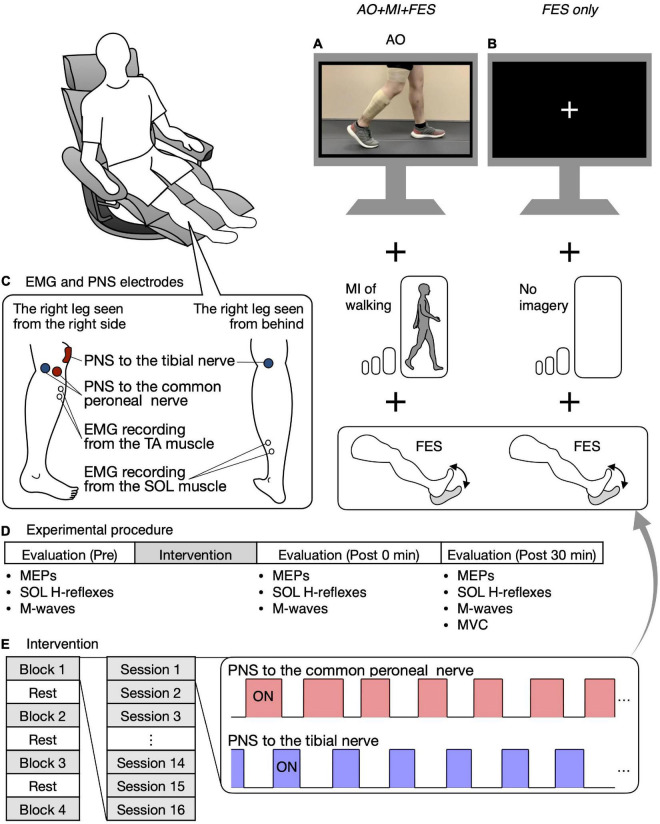
Study design and experimental procedure. Participants were seated in a chair placed in front of a 32-inch screen. In the *AO* + *MI* + *FES* condition, participants were asked to observe and imagine walking and were given electrical stimulation **(A)**. In the *FES only* condition, participants were asked to look at the center of the fixation cross and not imagine anything, while receiving given electrical stimulation as in the *AO* + *MI* + *FES* condition **(B)**. The position of electrodes for EMG recording from the TA and SOL muscles and for the common peroneal and tibial nerve stimulation are shown **(C)**. Before and after the intervention, MEPs, the recruitment curves of motor responses (M-waves and H-reflexes) in the SOL muscle, and Mmax were recorded **(D)**. The intervention consisted of four blocks and three breaks, with each block consisting of 16 20-s PNS sessions **(E)**. For each session, the PNS was given for 20 s at the timings corresponding to the EMG activities during actual walking.

Ten participants participated in two experiments, separated by an interval of at least 7 days [13.3 ± 4.8 days (7–21 days); mean ± SD, range in brackets]. We asked the participants to maintain the same level of their usual physical activity during the period between the two experiments. One of the experiments was performed in the *AO* + *MI* + *FES* condition, where the participants were asked to observe and imagine walking and were given PNS to the common peroneal and tibial nerves (Figure 2A). The other experiment was performed in the *only FES* condition, where the participants were asked to look at the center of a fixation cross and not to imagine anything and were given PNS as in the *AO* + *MI* + *FES* condition (Figure 2B). Participants initially took part in the experiment in the *AO* + *MI* + *FES* condition, then in the *only FES* condition. We investigated the effects of the intervention on corticospinal and spinal reflex excitability under these conditions. All procedures, except for the AO + MI part, were identical between conditions.

### Electromyographic recording

Figure 2C shows the position of the electrodes for the EMG recording. EMG signals were recorded from the right TA and SOL muscles. After cleaning the skin with alcohol, bipolar Ag/AgCl surface electrodes (Vitrode F-150S; Nihon Kohden, Tokyo, Japan) were placed over each muscle belly with at least 1 cm separation. A common reference electrode was placed around the knee. The EMG signals were amplified (×1,000) and filtered with a band-pass filter between 15 Hz and 1 kHz using a bio-amplifier system (MEG-6108; Nihon Kohden, Tokyo, Japan). The analog signals were digitized at a sampling rate of 4 kHz using an analog-to-digital converter (Powerlab/16SP, AD Instruments, Castle Hill, NSW, Australia).

### Stimulus settings

Two experiments in the *AO* + *MI* + *FES* and *FES only* conditions were conducted on different days. Stimulus setting such as position and intensity was individually set at each experiment since the positions of the EMG electrodes inevitably varied between experiments. The stimulus setting is explained in detail in the following sections “Transcranial magnetic stimulation” and “Peripheral nerve stimulation.”

#### Transcranial magnetic stimulation

Transcranial magnetic stimulation was applied over the primary motor cortex using a magnetic stimulator (Magstim 200, Magstim Co., Whitland, United Kingdom) that delivered monophasic pulses through a double-cone coil (external diameter: 110 mm; Magstim Co., Whitland, United Kingdom). The two optimal coil positions, or hotspots, for each of the TA and SOL muscles, were determined when the largest MEP amplitudes were elicited from each muscle. Once the hotspots were determined, they were used as targets using a TMS neuronavigation system (BrainSight, Rogue Research, Montreal, QC, Canada). The neuro-navigation system allowed the maintenance of accurate coil position over the hotspots throughout the experiments. A new search for the hotspot was performed in each experiment.

The resting motor thresholds (RMT) at the hotspots for the TA and SOL muscles were determined based on the guidelines outlined in a previous study (Rossini et al., 2015). The RMTs were defined as the minimum TMS intensity evoking MEPs with peak-to-peak amplitudes of at least 50 μV in each muscle at rest in at least five of ten successive trials. The RMTs for the TA muscle in the *AO* + *MI* + *FES* and *FES only* conditions corresponded to 38–63% (mean ± SD = 46.5 ± 7.4%) and 38–62% (mean ± SD = 45.7 ± 7.1%) of the maximum stimulator output, respectively. The RMTs for the SOL muscle in the *AO* + *MI* + *FES* and *FES only* conditions corresponded to 38–60% (mean ± SD = 46.4 ± 6.1%) and 38–66% (mean ± SD = 47.3 ± 7.3%) of the maximum stimulator output, respectively. No difference was observed in the RMTs between the *AO* + *MI* + *FES* and *FES only* conditions [TA, *t*_(9)_ = 0.739, *p* = 0.479; SOL, *t*_(9)_ = 0.876, *p* = 0.404, paired *t*-test]. The stimulation intensity for TMS was set to 120% of the RMT (1.2 RMT) in the evaluation of the effects of the interventions on corticospinal excitability.

#### Peripheral nerve stimulation

Constant current stimulation was delivered to the common peroneal and tibial nerves using two constant-current electrical stimulators (DS7A and DS7R, Digitimer, Welwyn Garden City, United Kingdom). Monophasic stimulus pulse duration was set to 1 ms for both the evaluation and intervention (Khaslavskaia and Sinkjaer, 2005; Lagerquist et al., 2012). Figure 2C shows the positions of the electrodes during PNS.

For common peroneal nerve stimulation, circular electrodes with a diameter of 3.2 cm (ValuTrode, Axelgaard, Fallbrook, CA, United States) were located near the fibular head, in the position that elicited the largest amplitude motor response (M-wave) in the TA muscle. The cathode and anode circular electrodes were placed at the back and front of the fibular head, respectively. For tibial nerve stimulation, a 5 × 5 cm square electrode (StimTrode, Axelgaard, Fallbrook, CA, United States) was placed over the patella as the anode. Then, a 3.2 cm diameter circular electrode (ValuTrode, Axelgaard, Fallbrook, CA, United States) was placed over the posterior tibial nerve at the popliteal fossa as the cathode, which induced the largest amplitude H-reflex in the SOL muscle. The electrodes were fixed with an adhesive tape.

For the intervention, common peroneal and tibial nerve stimulation intensities were set to induce a motor response (M-wave or H-reflex) with an amplitude greater than 100 μV and a visible twitch of the TA and SOL muscles (Khaslavskaia and Sinkjaer, 2005). For common peroneal nerve stimulation, some participants had difficulty in inducing H-reflexes, while H-reflexes could be induced for tibial nerve stimulation in all participants. Thus, in addition to a visible twitch, common peroneal and tibial nerve stimulation intensities were set based on M-waves and H-reflexes, respectively. If both common peroneal and tibial nerve stimulation intensities were determined by M-waves, the SOL twitch would be much greater than the TA twitch because of H-reflex near the maximum value associated with the M-wave in the SOL muscle. Therefore, M-waves and H-reflexes were used for setting PNS intensities in a mixed manner to match the degree of ankle plantar flexion and dorsiflexion. Thus, PNS with the intensities achieved functional ankle motion and induced sensory inputs to the cortex. We confirmed that PNS intensity was endurable for all participants. The stimulus intensities for common peroneal nerve PNS in the *AO* + *MI* + *FES* and *FES only* conditions were 2.5–11 mA (mean ± SD = 6.3 ± 2.7 mA) and 4–10 mA (mean ± SD = 6.4 ± 2.0 mA), respectively. The stimulus intensities for tibial nerve PNS in the *AO* + *MI* + *FES* and *FES only* conditions were 6–11 mA (mean ± SD = 8.0 ± 1.6 mA) and 5–13 mA (mean ± SD = 7.3 ± 2.4 mA), respectively. No differences were observed in the stimulus intensities for PNS between the *AO* + *MI* + *FES* and *FES only* conditions [TA, *t*_(9)_ = 0.176, *p* = 0.864; SOL, *t*_(9)_ = 1.709, *p* = 0.122, paired *t*-test]. The PNS pulse frequency was set to 30 Hz based on previous studies testing TA and SOL muscles FES (Khaslavskaia and Sinkjaer, 2005; Lagerquist et al., 2012).

### Experimental procedure

Participants sat in a chair in front of a 32-inch screen (697.7 × 392.3 mm, Multisync V321, NEC, Tokyo, Japan). The distance between the center of the chair and the screen was set to 1.5 m. The participants were asked to keep their bodies still and relax in a semi-sitting position (knee extension angle of 0° and hip flexion angle of 60°) during the entire experiment (Figure 2).

Before and after the intervention, MEPs in each recorded muscle, the recruitment curves of M-waves and H-reflexes in the SOL muscle only, and M-waves with the maximum amplitude (i.e., Mmax) in each recorded muscle were obtained at rest (Figure 2D). MEPs and H-reflexes reflect the excitability of the corticospinal pathway and the spinal reflex circuits, respectively. Mmax was recorded to normalize the H-reflexes. First, 15 MEPs were recorded from the TA and SOL muscles using the corresponding coil position and intensity for each muscle. Second, the recruitment curves of motor responses (M-wave and H-reflex) in the SOL muscle were obtained. The stimulus intensity was initially set to an intensity that elicited neither M-wave nor H-reflex and was gradually increased by 1 mA or 2 mA until the M-wave amplitude was greater than the H-reflex amplitude. Then, the stimulus intensity was increased by 5 mA or 10 mA until the M-wave maximum amplitude reached a plateau, that is, increasing the stimulus intensity no longer increased the size of the M-wave (i.e., Mmax). Three stimuli were provided for each intensity level. Lastly, we visually checked Mmax in the TA muscle with an oscilloscope and determined the stimulus intensity to induce it. The stimulus intensity was initially set to an intensity that elicited M-wave and was increased by 5 mA or 10 mA until the M-wave maximum amplitude reached a plateau. Mmax in the TA muscle was recorded using the determined intensity. Then, a band-pass filter between 150 Hz and 1 kHz was applied to reduce the stimulation artifact. The absence of changes in Mmax suggests a lack of changes in single fiber action potentials, which reflect parts of the peripheral fatigue effects (Gandevia, 2001). Thus, Mmax in the recorded muscles were measured to investigate the interventions’ peripheral fatigue effects.

Before the intervention, participants received different instructions according to the experimental condition. In the *AO* + *MI* + *FES* condition, the walking video synchronized with the FES (i.e., common peroneal and tibial nerve stimulation) was presented on the screen. The walking video and FES timing were controlled using MATLAB (2021a, The MathWorks Inc., Natick, MA, United States) and an analog output device (NI USB-6259, National Instruments, Austin, TX, United States). In the *AO* + *MI* + *FES* condition, participants were instructed to observe the walker’s right leg and to imagine that they were walking like the walker without performing voluntary contraction. The instruction was as follows: “*please observe his right leg and imagine that you are walking according to the observed stance and swing phases of walking without performing voluntary contraction*.” Subjects received a 1-min training session of AO + MI of walking. In the *FES only* condition, participants were asked to look at the center of a fixation cross presented on the screen. The instruction was as follows: “*please observe the center of the fixation cross without performing voluntary contraction nor imagining anything.”* The same instructions were provided to all participants.

The intervention in both conditions consisted of four blocks (Figure 2E). A break of at least 1 min was allowed between blocks. For each block, 16 20-s PNS sessions were performed at intervals of 6.5 s. For each session, common peroneal and tibial nerve stimulation was delivered for 20 s at the timings corresponding to EMG activities in the TA and SOL muscles during actual walking (Figure 2E). The timings were set as per Section “Determination of the on and off phases of electromyographic corresponding to the walking video.” One of the 16 sessions was a catch trial with no PNS to confirm that participants did not perform any voluntary TA and SOL muscles contractions. The order of the catch trials was randomized. In all catch trials, we visually confirmed that there was no muscle activity in any muscle in either condition.

In the *AO* + *MI* + *FES* condition, we confirmed that the participants could perform the motor imagery as we asked during the intervention by using a visual analog scale (VAS) (Moriuchi et al., 2020). After each block, participants were asked to make a mark on a 10 cm-VAS line on paper, which provided the VAS score. The left and right ends were labeled “*none at all*” (0 cm) and “*perfectly clear and vivid”* (10 cm), respectively.

Before and after the intervention, MEPs, recruitment curves of H-reflexes and M-waves, and Mmax at rest were obtained at 0 and 30 min. At the end of the experiment, EMG signals in the TA and SOL muscles were recorded for maximum voluntary contraction (MVC). Participants were asked to contract each muscle at maximal strength against manual resistance and hold this position for 3 s while the experimenter held their ankle to prevent them from moving.

### Data and statistical analyses

For the *AO* + *MI* + *FES* and *FES only* conditions, the peak-to-peak MEP amplitudes in the TA and SOL muscles were calculated offline using a custom-written script in MATLAB (2019b, The MathWorks Inc., Natick, MA, United States). The amplitudes were averaged for each participant at each time point (i.e., before, and 0 and 30 min after the intervention). The peak-to-peak amplitudes of the H-reflexes and M-waves in the SOL muscle were calculated and averaged for each participant and intensity. Then, the maximum amplitudes of the H-reflexes and M-waves (i.e., Hmax and Mmax) were obtained at each time point. H/Mmax was calculated by normalizing Hmax to Mmax. The peak-to-peak amplitudes of M-waves in the TA muscle were calculated and averaged for each participant at each time point. The average amplitudes of MEP, H/Mmax, and Mmax obtained after the intervention were normalized as percentage of the average amplitudes recorded before the intervention. The EMG root mean square (RMS) value of a 50-ms time window before TMS and PNS was defined as the background EMG activity for each muscle, and it was normalized according to the EMG activity for MVC. The RMS of the EMG signals measured for MVC was calculated for each 50-ms window, and the maximum RMS value was used as the MVC in each muscle.

All statistical analyses were performed using SPSS Statistics ver. 25 (IBM Corp., Chicago, IL, United States). First, statistical analyses were performed to investigate the effects of the intervention in each condition, that is, to compare the normalized MEP, Mmax, and H/Mmax before and after the intervention. Non-parametric tests were used because the Shapiro–Wilk tests showed that the normalized MEP, Mmax, and H/Mmax before the intervention (i.e., 100%) were not normally distributed. For the normalized amplitudes in each condition, the Friedman test, a non-parametric equivalent for a repeated-measure analysis of variance (rm-ANOVA), was conducted to compare the amplitudes before and after the intervention at each time point (i.e., before, 0 and 30 min after the intervention). If the Friedman tests showed a significant effect, Wilcoxon signed-rank tests were performed for multiple comparisons using *post hoc* tests.

Second, statistical analyses were performed to compare the effects of the intervention between the *AO* + *MI* + *FES* and *FES only* conditions, and between the TA and SOL muscles, that is, to compare the normalized MEP, Mmax, and H/Mmax after the intervention between times, between conditions, and for the MEP only between the muscles. Paired *t*-tests were conducted to compare the non-normalized MEP, Mmax, and H/Mmax before the intervention, as well as TMS and PNS intensity used in the intervention between the *AO* + *MI* + *FES* and *FES only* conditions. Parametric tests were used because the Shapiro–Wilk tests showed that the normalized MEP, Mmax, H/Mmax, and 0 and 30 min after the intervention were normally distributed. A three-way rm-ANOVA was performed to compare the changes in the normalized MEP [two muscles (the TA and SOL muscles), two conditions (the *AO* + *MI* + *FES* and *FES only* conditions), and two time points (0 and 30 min after the intervention)]. If the rm-ANOVA tests showed a significant main effect, *post hoc* tests for multiple comparisons were performed to identify significant differences. If the rm-ANOVA tests showed a significant interaction, subsequent two-way rm-ANOVAs were conducted. Next, two-way rm-ANOVAs were performed to compare the changes in the normalized Mmax and H/Mmax [two conditions (the *AO* + *MI* + *FES* and *FES only* conditions) and two time points (0 min and 30 min after the intervention)]. If the rm-ANOVA tests showed a significant main effect, *post hoc* tests for multiple comparisons were performed to identify significant differences. If the rm-ANOVA tests showed a significant interaction, simple main effect tests were conducted to identify the source of the interaction.

Lastly, statistical analyses were performed to confirm that there was no change in background EMG activity before TMS and PNS (i.e., MEPs and Hmax were induced). Two-way rm-ANOVAs were performed to compare the changes in the background EMG activity normalized with MVC between the two conditions [(the *AO* + *MI* + *FES* and *FES only* conditions) and three time points (before, 0 and 30 min after the intervention)]. In case of a significant violation of the assumption of sphericity (Mauchly’s test, *p* < 0.05), Greenhouse–Geisser adjustments to the degrees of freedom were performed. The subsequent statistical procedures were the same as those performed for the changes in the normalized MEP and H/M, which are described above.

The significance level was set to 0.05 in all statistical tests, and the Holm method was used to correct *p*-Values for multiple comparisons. The eta squared values for Friedman tests and ANOVA tests, *r*-values for Wilcoxon signed-rank tests, and *d*-values for paired t-tests were calculated as the effect size indices (Cohen, 1988; Rosenthal et al., 1994; Morse, 1999). The thresholds for interpreting the eta squared values were set to 0.01, 0.06, and 0.14 for small, medium, and large, respectively, while those for interpreting the *r*-values were set at 0.1, 0.3, and 0.5, for small, medium, and large, respectively (Cohen, 1988; Rosenthal et al., 1994; Morse, 1999). Data are presented as mean ± SD.

## Results

### Motor evoked potential, Mmax, and H/Mmax

**FIGURE 3:**
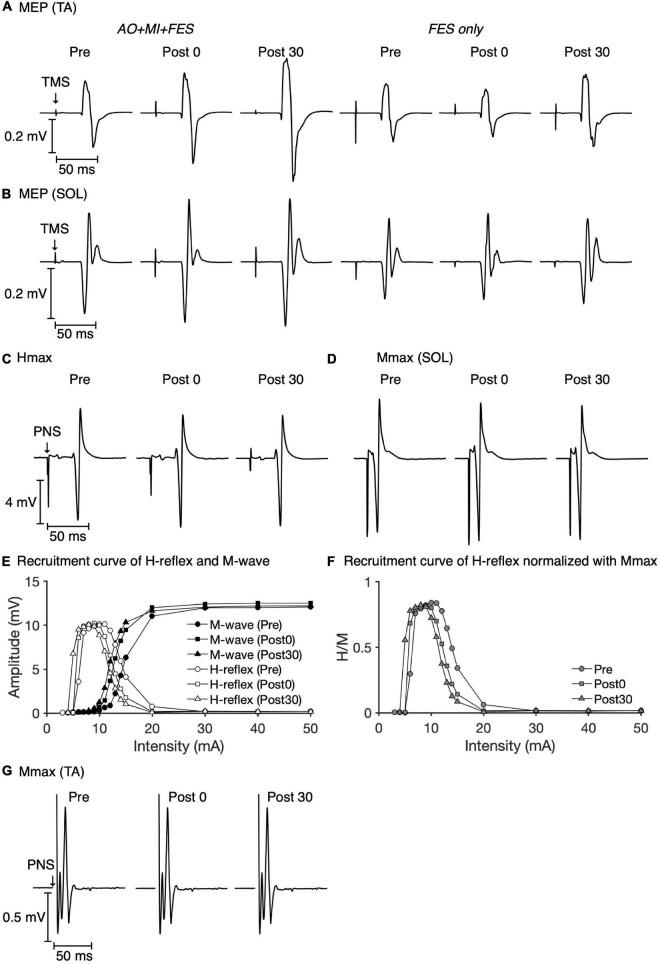
Examples of MEP, Hmax, and Mmax waveforms and recruitment curves recorded from one participant. The mean MEP waveforms in the TA **(A)** and SOL muscles **(B)** recorded at each time point in each condition are shown. The mean waveforms of Hmax **(C)** and Mmax **(D)** in the SOL muscle at each time point in the *AO* + *MI* + *FES* condition are shown. Recruitment curves of H-reflex, M-wave **(E)**, and H-reflex normalized with Mmax **(F)** at each time point in the *AO* + *MI* + *FES* condition are shown. The mean waveforms of Mmax in the TA muscle **(G)** at each time point in the *AO* + *MI* + *FES* condition are shown.

Figure 3 represents the mean waveforms of MEP, Hmax, and Mmax and recruitment curves of H-reflex, M-wave, and H-reflex normalized to Mmax recorded from one participant. Table 1 shows the average non-normalized and normalized amplitudes of MEP, Mmax, Hmax, and H/Mmax with SDs.

**TABLE 1:** The average non-normalized and normalized amplitudes of MEP, Mmax, Hmax, and H/Mmax with SDs.

		Non-normalized amplitudes (mV)						Normalized amplitudes (%)					
			
		*AO* + *MI* + *FES*			*FES only*			*AO* + *MI* + *FES*			*FES only*		
MEP (TA)	Pre	0.215	±	0.145	0.233	±	0.143	100	±	0	100	±	0
	Post 0	0.305	±	0.240	0.241	±	0.184	146	±	71	99.2	±	43.5
	Post 30	0.495	±	0.242	0.302	±	0.196	283	±	148	132	±	52
MEP (SOL)	Pre	0.186	±	0.118	0.172	±	0.092	100	±	0	100	±	0
	Post 0	0.184	±	0.119	0.159	±	0.112	111	±	45	91.7	±	27.7
	Post 30	0.208	±	0.135	0.191	±	0.130	118	±	33	107	±	27
Mmax (TA)	Pre	1.61	±	0.39	1.65	±	0.49	100	±	0	100	±	0
	Post 0	1.62	±	0.43	1.64	±	0.47	101	±	6	100	±	14
	Post 30	1.69	±	0.46	1.71	±	0.48	105	±	7	105	±	10
Mmax (SOL)	Pre	10.7	±	1.6	11.9	±	2.4	100	±	0	100	±	0
	Post 0	10.9	±	1.6	11.6	±	2.1	102	±	8	97.7	±	4.8
	Post 30	10.9	±	1.6	11.7	±	2.2	102	±	11	99.2	±	6.8
Hmax (SOL)	Pre	4.51	±	3.29	5.29	±	2.32	100	±	0	100	±	0
	Post 0	4.54	±	3.21	4.96	±	2.34	103	±	11	94.0	±	13.6
	Post 30	4.37	±	2.67	4.95	±	2.06	107	±	26	95.9	±	13.7
H/Mmax (SOL)	Pre	0.399	±	0.240	0.443	±	0.183	100	±	0	100	±	0
	Post 0	0.396	±	0.229	0.429	±	0.193	100	±	8	96.5	±	14.2
	Post 30	0.393	±	0.201	0.423	±	0.172	104	±	18	96.7	±	12.9

**FIGURE 4:**
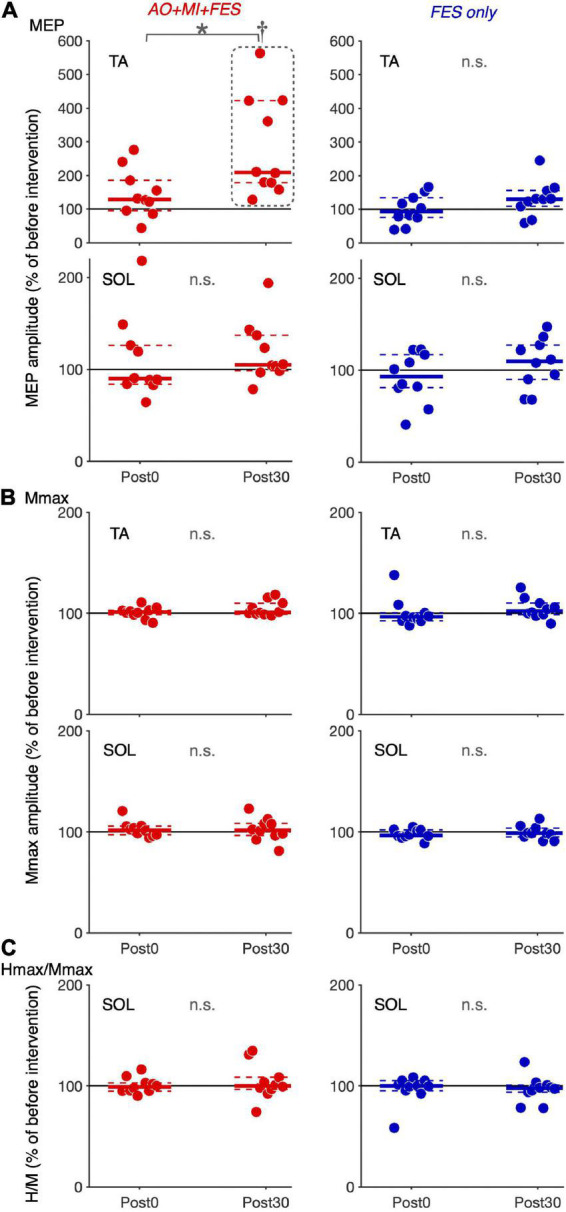
Changes in the average amplitudes of MEP, Mmax, and H/Mmax after intervention. Plot graphs showing the mean changes in average MEP amplitudes **(A)**, Mmax **(B)**, and H/Mmax **(C)** after the intervention (Post0 and Post30) in the *AO* + *MI* + *FES* (red) and *FES only* (blue) conditions. Each plot displays the average values normalized as percentage of the baseline before intervention. Colored solid and dashed lines represent median and interquartile ranges, respectively. Black lines indicate 100%. An asterisk (*) and dagger (†) indicate significant differences (Holm-corrected *p*-value < 0.05). Friedman and Wilcoxon signed-rank tests revealed that in the TA muscle, MEP amplitudes significantly increased, 30 min after intervention in the *AO* + *MI* + *FES* condition, compared to before (†) and 0 min after intervention (*). Friedman tests revealed that there were no significant effects regarding time point for Mmax in each muscle, and for MEP and H/Mmax in the SOL muscle.

Friedman tests revealed significant differences in the normalized MEP between time points in the *AO* + *MI* + *FES* condition in the TA muscle [χ^2^ (2) = 15.80, *p* < 0.001, η^2^ = 1.580], but not in the SOL muscle [Figure 4A; χ^2^ (2) = 2.600, *p* = 0.273, η^2^ = 0.260]. There were no significant differences in the normalized MEP for both muscles in the *FES* condition [Figure 4A; TA, χ^2^ (2) = 3.800, *p* = 0.150, η^2^ = 0.380; SOL, χ^2^ (2) = 1.400, *p* = 0.497, η^2^ = 0.140]. There were no significant differences in the normalized Mmax for both muscles in either condition [TA, *AO* + *MI* + *FES*, χ^2^ (2) = 3.200, *p* = 0.202, η^2^ = 0.320; TA, *FES only*, χ^2^ (2) = 4.200, *p* = 0.122, η^2^ = 0.420; SOL, *AO* + *MI* + *FES*, χ^2^ (2) = 0.800, *p* = 0.670, η^2^ = 0.080; SOL, *FES only*, χ^2^ (2) = 1.400, *p* = 0.497, η^2^ = 0.140], nor in the normalized H/Mmax in either condition [Figures 4B,C; *AO* + *MI* + *FES*, χ^2^ (2) = 0.200, *p* = 0.905, η^2^ = 0.020; *FES only*, χ^2^ (2) = 0.800, *p* = 0.670, η^2^ = 0.080]. Wilcoxon signed-rank tests revealed that, in the *AO* + *MI* + *FES* condition, the normalized MEP amplitudes in the TA muscle significantly increased 30 min after the intervention, compared to those before and 0 min after the intervention [Figure 4A; Pre vs. Post 30, *z* = 2.803, Holm-corrected *p* = 0.015, *r* = 0.886; Post 0 vs. Post 30, *z* = 2.803, Holm-corrected *p* = 0.015, *r* = 0.886]. There was no difference in the normalized MEP amplitudes before and 0 min after the intervention (*z* = 1.784, Holm-corrected *p* = 0.077, *r* = 0.564).

**FIGURE 5:**
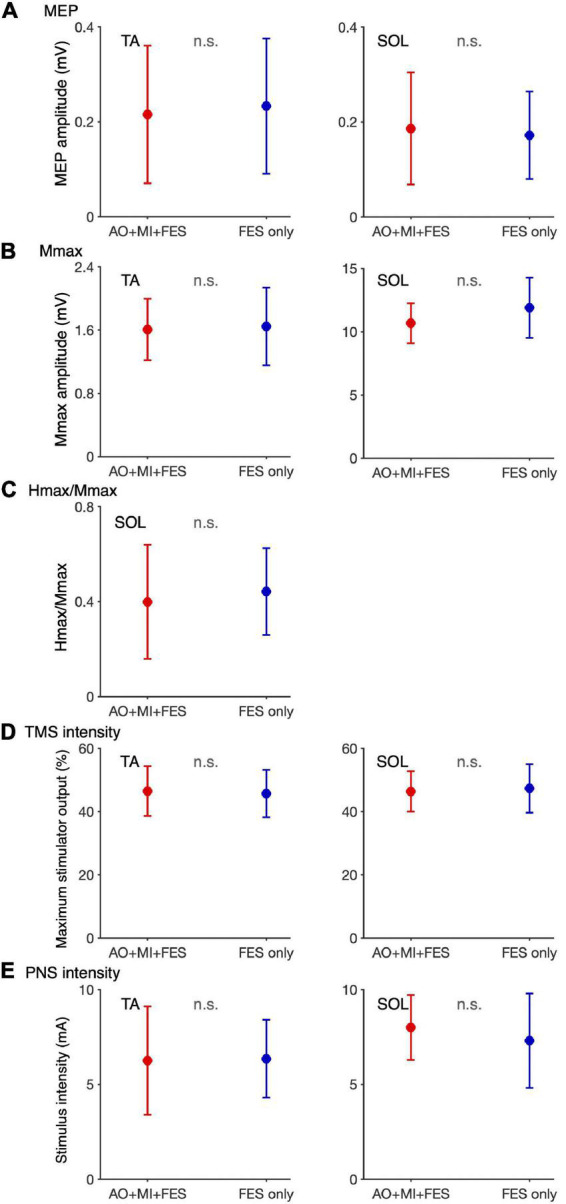
Comparison of the non-normalized amplitudes of MEP, H/Mmax recorded before the intervention and TMS and PNS intensities between the *AO* + *MI* + *FES* and *FES only* conditions. Plots show the averages of non-normalized MEP **(A)** and Mmax **(B)** amplitudes and H/Mmax **(C)** recorded before intervention and TMS **(D)** and PNS **(E)** intensities in the *AO* + *MI* + *FES* (red) and *FES only* (blue) conditions. Error bars represent the standard deviation. Paired *t*-test showed no significant differences in the non-normalized amplitudes and intensities between the conditions for both muscles.

We compared the non-normalized amplitudes recorded before the intervention and TMS and PNS intensities between the *AO* + *MI* + *FES* and *FES only* conditions (Figure 5). Paired *t*-test showed no significant differences in the non-normalized amplitudes and intensities between the conditions for both muscles [TA, MEP, *t*(9) = 0.680, *p* = 0.514, *d* = 0.129; TA, Mmax, *t*(9) = 0.220, *p* = 0.831, *d* = 0.095; TA, TMS, *t*(9) = 0.739, *p* = 0.479, *d* = 0.110; TA, PNS, *t*(9) = 0.176, *p* = 0.864, *d* = 0.042; SOL, MEP, *t*(9) = 0.627, *p* = 0.546, *d* = 0.142; SOL, Mmax, *t*(9) = 1.215, *p* = 0.255, *d* = 0.605, SOL, H/Mmax, *t*(9) = 1.325, *p* = 0.218, *d* = 0.215, SOL, TMS, *t*(9) = 0.876, *p* = 0.404, *d* = 0.134; SOL, PNS, *t*(9) = 1.709, *p* = 0.122, *d* = 0.340].

**FIGURE 6:**
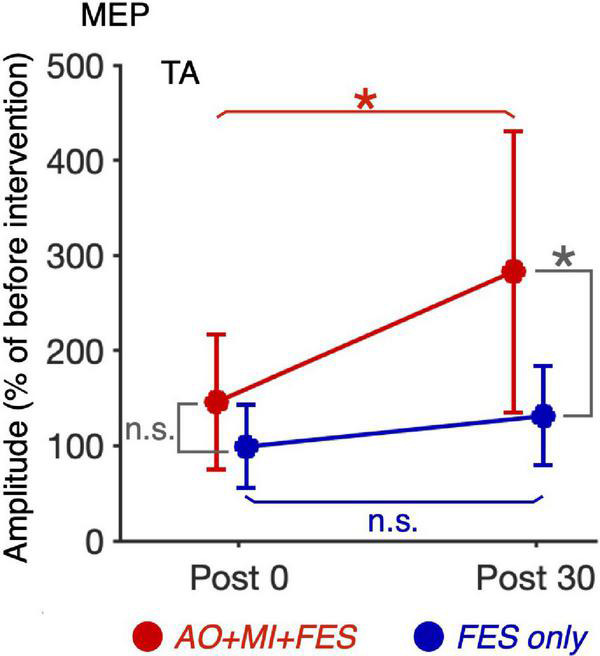
Comparison of the changes in the average MEP amplitudes in the TA muscle after intervention between the *AO* + *MI* + *FES* and *FES only* conditions. Line graphs show mean changes in average MEP amplitudes in the TA muscle normalized as percentage of the baseline before intervention in the *AO* + *MI* + *FES* (red) and *FES only* (blue) conditions. Error bars represent the standard deviation. ANOVA and paired *t*-tests revealed that in the TA muscle, MEP amplitudes significantly increased 30 min after intervention in the *AO* + *MI* + *FES* condition, but not in the *FES only* condition, compared to 0 min after intervention. Additionally, the changes in MEP 30 min after intervention were greater in the *AO* + *MI* + *FES* condition than in the *FES only* condition. * and n.s. indicate significant differences (Holm-corrected *p*-value < 0.05) and no significant differences, respectively.

**FIGURE 7:**
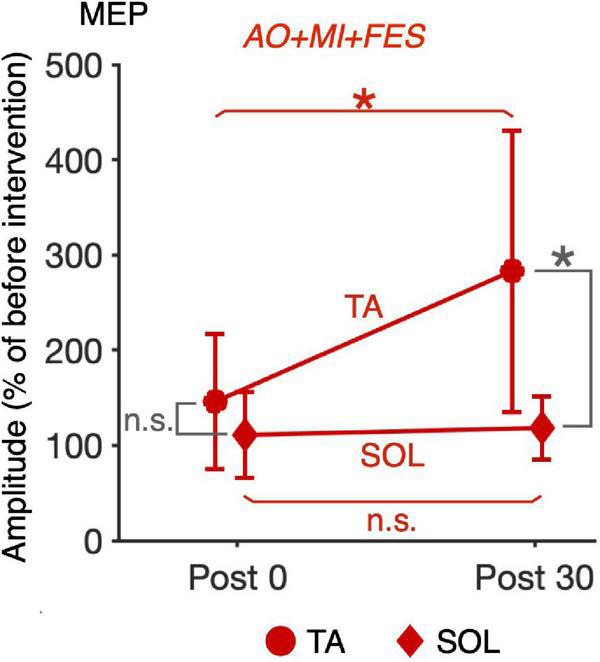
Comparison of the changes in the average MEP amplitudes after intervention between the TA and SOL muscles in the *AO* + *MI* + *FES* condition. Line graphs show mean changes in average MEP amplitudes normalized as percentage of the baseline before intervention in the *AO* + *MI* + *FES* condition. Error bars represent the standard deviation. Circles and diamonds indicate TA and SOL, respectively. Asterisks indicate significant differences (Holm-corrected *p*-value < 0.05). ANOVA and paired *t*-tests revealed that in the TA muscle, but not in the SOL muscle, MEP amplitudes significantly increased 30 min after intervention in the AO + MI + FES condition, compared to 0 min after intervention. Additionally, ANOVA and paired *t*-tests revealed that in the AO + MI + FES condition, the changes in MEP 30 min after intervention were greater in the TA muscle than in the SOL muscle. * and n.s. indicate significant differences (Holm-corrected *p*-value < 0.05) and no significant differences, respectively.

For the normalized MEP in the TA and SOL muscles, a three-way rm-ANOVA showed a significant three-factor interaction [second-order interaction, *F*(1, 9) = 9.423, *p* = 0.013*, η^2^ = 0.512]. Two-way rm-ANOVAs (condition × time point) revealed a significant main effect of time point [*F*(1, 9) = 14.92, *p* = 0.004, η^2^ = 0.624], not main effect of condition [*F*(1, 9) = 6.785, *p* = 0.290, η^2^ = 0.430] and interaction for the normalized MEP in the TA muscle [Figure 6; *F*(1, 9) = 9.828, *p* = 0.012, η^2^ = 0.522]. The two-way rm-ANOVAs did not reveal significant main effects involving condition [*F*(1, 9) = 1.693, *p* = 0.226, η^2^ = 0.158], time point [*F*(1, 9) = 4.444, *p* = 0.064, η^2^ = 0.331], or their interaction for the normalized MEP in the SOL muscle [*F*(1, 9) = 2.416, *p* = 0.155, η^2^ = 0.212]. *Post hoc* tests revealed that, in the *AO* + *MI* + *FES* condition, the normalized MEP amplitudes in the TA muscle 30 min after the intervention were significantly greater than those of 0 min after the intervention [Figure 6; *t*(9) = 3.746, *p* = 0.018, *d* = 1.056, paired *t*-test], but not in the *FES only* condition [Figure 6; *t*(9) = 2.415, *p* = 0.078, *d* = 0.670, paired *t*-test]. Moreover, the normalized MEP amplitudes in the TA muscle 30 min after the intervention, but not 0 min after the intervention, in the *AO* + *MI* + *FES* condition were significantly greater than those in the *FES only* condition [Figure 6; 0min, *t*(9) = 1.438, *p* = 0.184, *d* = 0.776; 30min, *t*(9) = 3.100, *p* = 0.038, *d* = 1.169]. Two-way rm-ANOVAs (muscle × time point) revealed a significant main effect of muscle [*F*(1, 9) = 9.457, *p* = 0.002, η^2^ = 0.512] and time [*F*(1, 9) = 17.45, *p* = 0.013, η^2^ = 0.660] and interaction [Figure 7; *F*(1, 9) = 10.91, *p* = 0.009, η^2^ = 0.548] for the *AO* + *MI* + *FES* condition, as well as a significant main effect of time point for the *FES only* condition [*F*(1, 9) = 8.465, *p* = 0.017, η^2^ = 0.485]. The two-way rm-ANOVAs did not reveal significant main effects involving muscle point [*F*(1, 9) = 1.745, *p* = 0.219, η^2^ = 0.162] or interaction [*F*(1, 9) = 1.592, *p* = 0.239, η^2^ = 0.150] for the *FES only* condition. Comparing muscles in the *AO* + *MI* + *FES* condition, *post hoc* tests revealed that the normalized MEP amplitudes in the TA muscle 30 min after the intervention, but not 0 min after the intervention, were significantly greater than those in the SOL muscle [Figure 7; 0 min, *t*(9) = 1.221, *p* = 0.253, *d* = 0.597, 30 min, *t*(9) = 3.636, *p* = 0.016, *d* = 1.259, paired *t*-test]. *Post hoc* tests did not reveal significant differences in the normalized MEP amplitudes in the SOL muscle between 0 min and 30 min after the intervention [Figure 7; *t*(9) = 1.339, *p* = 0.427, *d* = 0.193, paired *t*-test]. For the normalized Mmax in the TA and SOL muscles and H/Mmax, the two-way rm-ANOVAs did not reveal significant main effects involving condition [TA, Mmax, *F*(1, 9) = 0.001, *p* = 0.973, η^2^ < 0.001; SOL, Mmax, *F*(1, 9) = 3.326, *p* = 0.101, η^2^ = 0.270; SOL, H/Mmax, *F*(1, 9) = 1.274, *p* = 0.288, η^2^ = 0.124], time point [TA, Mmax, *F*(1, 9) = 5.018, *p* = 0.052, η^2^ = 0.358; SOL, Mmax, *F*(1, 9) = 0.289, *p* = 0.604, η^2^ = 0.031; SOL, H/Mmax, *F*(1, 9) = 0.143, *p* = 0.714, η^2^ = 0.016], or their interaction [TA, Mmax, *F*(1, 9) = 0.009, *p* = 0.925, η^2^ = 0.001;SOL, Mmax, *F*(1, 9) = 0.242, *p* = 0.634, η^2^ = 0.026; SOL, H/Mmax, *F*(1, 9) = 1.061, *p* = 0.330, η^2^ = 0.105].

### Background electromyographic activity and visual analog scale scores

Table 2 shows the average non-normalized and normalized background EMG activity before TMS and PNS (i.e., MEPs and Hmax were induced), and MVC as RMS values (Table 2). Background EMG activity before PNS was greater than before TMS, probably due to the power supply noise of the electrical stimulator. For the normalized background EMG activity before TMS and PNS, two-way rm-ANOVAs (condition × time point) did not reveal significant main effects involving condition [TA, TMS, *F*(1, 9) = 0.152, *p* = 0.705, η^2^ = 0.017; SOL, TMS, *F*(1, 9) = 1.724, *p* = 0.222, η^2^ = 0.161; SOL, PNS, *F*(1, 9) = 0.815, *p* = 0.390, η^2^ = 0.083], time point [TA, TMS, *F*(1, 9) = 2.242, *p* = 0.135, η^2^ = 0.199; SOL, TMS, *F*(1, 9) = 2.264, *p* = 0.157, η^2^ = 0.201; SOL, PNS, *F*(1, 9) = 1.096, *F*(1, 9) = 1.096, η^2^ = 0.109], or their interaction [TA, TMS, *F*(1, 9) = 0.958, *p* = 0.402, η^2^ = 0.096; SOL, TMS, *F*(1, 9) = 1.433, *p* = 0.265, η^2^ = 0.137; SOL, PNS, *F*(1, 9) = 1.109, *p* = 0.351, η^2^ = 0.110].

**TABLE 2:** The average non-normalized and normalized background EMG activity and MVC.

		Non-normalized RMS value (μ V)						Normalized RMS value (%MVC)					
			
		*AO* + *MI* + *FES*			*FES only*			*AO* + *MI* + *FES*			*FES only*		
Background EMG before TMS (TA)	Pre	1.84	±	0.46	1.84	±	0.45	0.387	±	0.159	0.371	±	0.172
	Post 0	1.81	±	0.26	1.87	±	0.32	0.389	±	0.165	0.368	±	0.142
	Post 30	1.97	±	0.38	1.84	±	0.40	0.418	±	0.164	0.371	±	0.178
Background EMG before TMS (SOL)	Pre	2.06	±	0.64	2.02	±	0.57	0.516	±	0.161	0.645	±	0.261
	Post 0	2.45	±	0.18	2.11	±	0.10	0.593	±	0.324	0.638	±	0.258
	Post 30	2.22	±	0.64	3.12	±	0.22	0.568	±	0.205	0.986	±	0.849
Background EMG before PNS (SOL) for Hmax	Pre	8.98	±	5.76	10.5	±	17.6	2.42	±	1.66	2.64	±	2.98
	Post 0	10.7	±	8.26	15.7	±	22.7	2.89	±	2.59	4.22	±	4.68
	Post 30	9.49	±	11.43	12.9	±	14.7	2.58	±	3.40	3.95	±	3.92
MVC	TA	538	±	219	580	±	232		-			-	
	SOL	415	±	115	350	±	134		-			-	

In the *AO* + *MI* + *FES* condition, the average VAS scores with SDs measured after the first, second, third, and fourth block were 7.20 ± 0.91, 7.55 ± 1.25, 8.10 ± 0.91, and 7.94 ± 1.12, respectively.

## Discussion

The present study investigated the effects of FES intervention for ankle dorsiflexion and plantar flexion simultaneously with and without AO + MI of walking on corticospinal and spinal reflex excitability. MEP amplitudes in the TA muscle increased 30 min after the intervention in the *AO* + *MI* + *FES* condition (*p* < 0.05), but not in the *FES only* condition (*p* > 0.05, Figure 4A). MEP amplitudes and H-reflex (H/Mmax) in the SOL muscle did not change after the intervention in either condition (*p* > 0.05, Figures 4A,C). In the *AO* + *MI* + *FES* condition, the average VAS scores for each block were above 7 out of 10, suggesting that participants could perform MI of walking during the intervention. Our results demonstrated that the intervention combining AO + MI with FES facilitated corticospinal excitability in the TA muscle. We confirmed no EMG activity in catch trials during the interventions, which indicated that the participants did not perform voluntary contractions. Furthermore, there were no significant differences in M-waves (*p* > 0.05, Figure 4B) and in the background EMG activity before TMS and PNS in each measurement (*p* > 0.05) between time points and between conditions. These results indicate that the peripheral fatigue effects induced by FES and EMG activity are unlikely to influence our measurements. The following discussion explains the effects of the FES interventions with and without AO + MI.

### Facilitation of corticospinal excitability in the tibialis anterior muscle 30 min after the intervention combining AO + MI of walking with functional electrical stimulation

AO + MI of walking induces phase-dependent activation of the sensorimotor cortex similar to neural activity during walking (Yokoyama et al., 2020, 2021; Kaneko et al., 2021). The present study combined AO + MI of walking with FES based on walking phase-dependent EMG activity. The FES consisted of PNS with intensities above motor threshold that induced sensory inputs from sensory and cutaneous nerves and muscle contraction. Thus, the *AO* + *MI* + *FES* condition (i.e., the concurrent combination of AO + MI and FES) would synchronize cortical activation during AO + MI of walking with the sensory inputs induced by FES similarly according to walking phases. In this condition, facilitation of corticospinal excitability in the TA muscle was observed 30 min after the intervention (*p* < 0.05), while no facilitation resulted in the *FES only* condition (*p* > 0.05, Figure 4A). Because the intervention using only AO + MI of walking did not change corticospinal excitability (Kaneko et al., 2022), our results suggest that the facilitation was driven by the walking phase-dependent synchronization of cortical activation during AO + MI and sensory inputs induced by FES. This is supported by previous studies reporting that the timing of cortical activation and sensory inputs is an essential factor in facilitating corticospinal excitability (Stefan, 2000; Mrachacz-Kersting et al., 2007). Furthermore, a previous study showed that the synchronization of cortical activation during MI and sensory inputs plays an important role in plastic changes in corticospinal excitability (Mrachacz-Kersting et al., 2012).

Functional electrical stimulation during walking, sensory inputs from ankle dorsiflexion and plantar flexion interact with cortical activation to induce neural plastic changes (Kido Thompson and Stein, 2004; Khaslavskaia and Sinkjaer, 2005; Lagerquist et al., 2012). We propose that such plastic changes are related to the facilitation of corticospinal excitability induced by AO + MI combined with FES in the present study. A previous study reported that FES applied to the common peroneal nerve innervating the TA muscle (i.e., TA muscle FES) during walking facilitated corticospinal excitability in the TA muscle, and this facilitation lasted for at least 30 min (Kido Thompson and Stein, 2004). In addition, facilitation of corticospinal excitability in the TA muscle was observed at 0, 15, and 30 min after TA muscle FES during voluntary dorsiflexion (Khaslavskaia and Sinkjaer, 2005). Conversely, corticospinal excitability in the TA muscle decreased 0 min after FES applied to the tibial nerve innervating the SOL (i.e., SOL muscle FES) during voluntary plantar flexion, but not after 15 and 30 min (Khaslavskaia and Sinkjaer, 2005). We consider that the activation of the sensorimotor cortex induced by AO + MI partially resembles the activation during actual walking and voluntary dorsi/plantar flexion. Thus, when using FES for both the TA and SOL muscles, SOL muscle FES during voluntary plantar flexion temporally masks facilitatory effect of TA muscle FES on corticospinal excitability for the TA muscle during walking and voluntary dorsiflexion. This would explain the delayed facilitation of corticospinal excitability in the TA muscle we reported, that is, the AO + MI + FES condition did not change corticospinal excitability in the TA muscle 0 min after the intervention, while it facilitated the excitability 30 min after the intervention (*p* < 0.05, Figure 4A), when the inhibitory effect of SOL muscle FES during AO + MI disappeared.

### Differences in corticospinal excitability between the tibialis anterior and soleus muscles after the intervention in the *AO + MI + FES* condition

Our results showed that AO + MI combined with FES facilitated corticospinal excitability in the TA muscle, but not in the SOL muscle (*p* < 0.05, Figure 4A). The present study used the same criteria for TMS and PNS settings (e.g., stimulus intensity) in the TA and SOL muscles. Hence, our results suggest that the transient changes in corticospinal excitability in the TA muscle were greater than those in the SOL muscle. Specificity for these muscles was also observed after FES combined with voluntary ankle flexion (Khaslavskaia and Sinkjaer, 2005; Lagerquist et al., 2012). TA muscle FES during voluntary dorsiflexion facilitated corticospinal excitability in the TA muscle (Khaslavskaia and Sinkjaer, 2005), but SOL muscle FES during voluntary plantar flexion did not change the excitability in the SOL muscle (Lagerquist et al., 2012). Muscle specificity might be caused by greater cortical involvement in the TA muscle activation than in the SOL muscle activation. Moreover, the TA muscle might be more sensitive to sensory inputs than the SOL muscle at the cortical level, as the stretch reflex in the TA muscle has a clearer transcortical component than in the SOL muscle (Christensen et al., 2000).

### No changes in spinal reflex excitability after the intervention in the *AO + MI + FES* condition

A previous study reported that SOL muscle FES during plantar flexion increased spinal reflex excitability in the SOL muscle (Lagerquist et al., 2012). However, TA muscle FES during actual plantar flexion and MI increased reciprocal inhibition from the TA muscle to the SOL muscle (Takahashi et al., 2017, 2019). Because AO + MI combined with FES in the present study included components of both TA and SOL muscles FES, increases in reciprocal inhibition would counteract those in the spinal reflex. In addition to reciprocal inhibition, presynaptic inhibition mechanisms may also be involved. Presynaptic inhibition of SOL H-reflex increases after repetitive tibial nerve stimulation (Grosprêtre et al., 2018) but conversely decreases after MI training (Grosprêtre et al., 2019). Therefore, even if FES and AO + MI alone modulated presynaptic inhibition in the present study, they may cancel each of their modulations and not change spinal reflex excitability in the AO + MI + FES condition. Thus, reciprocal inhibition and presynaptic inhibition may underlie failure to change spinal reflex excitability in the *AO* + *MI* + *FES* condition (*p* > 0.05, Figure 4C). Alternatively, FES alone may be insufficient to modulate reciprocal inhibition and presynaptic inhibition due to the lack of H-reflex changes in the *FES alone* condition.

Another possibility is that resistance to transient changes in spinal reflex is caused by the lack of synchronization at the spinal level. In the present study, the FES timing synchronized with the EMG activity of the walker in the video depended on the walking phase. The AO + MI of walking facilitates spinal reflex excitability, but the facilitation does not depend on the walking phases (Kaneko et al., 2018, 2019). It is possible that the activation at the spinal level induced by AO + MI did not interact with sensory inputs induced by FES, and thus AO + MI combined with FES could not induce transient spinal changes.

### No changes in corticospinal and spinal reflex excitability after the intervention in the functional electrical stimulation only condition

Our results showed no modulation of corticospinal and spinal reflex excitability after a 20-min intervention in the *FES only* condition (*p* > 0.05, Figures 4A,C). Previous studies have reported that corticospinal excitability increases in the TA muscle and decreases in the SOL muscle after 30-min common peroneal nerve stimulation (Khaslavskaia et al., 2002; Knash et al., 2003; Khaslavskaia and Sinkjaer, 2005). However, another study showed that 20-min PNS was insufficient to modulate excitability (Takahashi et al., 2019). Furthermore, common peroneal and tibial nerves PNS did not change spinal reflex excitability in the TA and SOL muscles in several reports (Knash et al., 2003; Lagerquist et al., 2012). Therefore, a 20-min intervention using FES for both the TA and SOL muscles might not have been sufficient to induce transient changes at the cortical and spinal levels. In other words, AO + MI may improve the efficacy of FES on modulating corticospinal excitability and FES of 30 mins or longer may change the excitability.

### Limitations

The present study has several noteworthy limitations. First, this study measured corticospinal and spinal excitability but not cortical activity. We deductively assume a transient facilitation of cortical activity after the *AO* + *MI* + *FES* condition because there was facilitation of corticospinal excitability and no change in spinal excitability. However, measurements of cortical activity/excitability using electroencephalography and paired-pulse TMS paradigm are needed to achieve a more detailed picture of the effects of FES combined with AO + MI. Second, this study had a small sample size and recruited healthy individuals, not individuals with neurological gait deficits. For the facilitatory effect of *AO + MI + FES* on corticospinal excitability in the TA muscle, the effect sizes for Friedman tests, ANOVA tests, Wilcoxon signed-rank tests, and paired *t*-tests were greater than the criterion values for a large effect. Thus, *AO + MI + FES* would have a significant facilitatory effect on corticospinal excitability. Furthermore, although corticospinal tract excitability, which is associated with the recovery of gait after incomplete spinal cord injury (Thomas and Gorassini, 2005), was measured, gait functions such as walking speed were not recorded. Therefore, the results of this study do not provide direct evidence that FES combined with AO + MI improves gait function after neurological gait deficits. Third, the FES combined with AO + MI in this study would not be expected to engage the central pattern generating networks. This is a clear difference between FES combined with AO + MI and walking, indicating that AO + MI is unlikely to be a complete replacement for walking in this context. Fourth, we used a walking speed of 1.0 m/s, which is slow for healthy adults, when recording a video of a male walking for AO + MI. We determined the speed to make it easier to distinguish gait phase and muscle activity timing during AO + MI. In addition, using slow walking for AO and MI would be more suitable than using fast walking because AO and MI are used in the rehabilitation of patients suffering from neurological gait dysfunction. However, movement speed would influence neural activity (Tazoe and Perez, 2013; Iwane et al., 2019), which suggests neural activity can be affected by the walking speed of AO and MI. Thus, further studies are needed to investigate the effects of AO and MI on neural activity depending on the speed of the movement observed or imaged. Fifth, the FES timing corresponded to EMG activities in the TA and SOL when one participant, who did not take part in the experiment, was walking. However, the EMG activities of the walker in the walking video could differ from those of the participants, indicating that perfect synchronization of FES and AO + MI may not be possible. To solve this problem, the muscle activity during walking of each participant should be recorded in advance, and the FES timing should be determined based on the recorded activity. Individually determined FES timing may promote the effects of *AO + MI + FES* on neural activity. Sixth, we could not directly confirm that participants were relaxed and not voluntarily contracting the muscles when FES was applied. This is because the motor responses and power supply noise induced by FES made it difficult to confirm the EMG activity. As we could alternatively do, we set a catch trial in which PNS was not given for one of the 16 sessions and visually confirmed that there was no muscle activity in the recorded muscle in either condition. Thus, there would be no anticipatory muscle contraction or unconscious muscle activity. However, a possibility that participants contracted the muscles when FES was applied cannot be ruled out.

## Conclusion

Intervention using FES only for dorsiflexion and plantar flexion did not change corticospinal excitability, while synchronizing FES with AO + MI of walking facilitated excitability. Thus, synchronization of sensory inputs from FES and cortical activity during AO + MI would facilitate corticospinal excitability. Furthermore, facilitation was observed in the dorsiflexor muscle but not in the plantar flexor muscle, suggesting a muscle specificity effect in the intervention. These results demonstrate the transient effects of FES combined with AO + MI on corticospinal excitability.

## Data availability statement

The data presented in this manuscript were newly acquired for the present study. Due to data privacy concerns, they are not available to the community in open repositories. The datasets generated in this present study are available from the corresponding author upon reasonable request. In such cases, the reason for the data request and procedures for ensuring privacy will be reviewed and discussed.

## Ethics statement

The studies involving human participants were reviewed and approved by the local Ethics Committee of the University of Tokyo. The participants provided their written informed consent to participate in this study.

## Author contributions

NK contributed to the conceptualization, methodology, formal analysis, investigation, writing the original draft, and funding acquisition. AS contributed to the methodology, investigation, and writing—review and editing. HY contributed to the conceptualization, methodology, investigation, writing—review and editing, and supervision. YM contributed to the methodology and writing—review and editing. KN wrote, reviewed, and edited the manuscript, supervised the data, and carried out the funding acquisition. All authors contributed to the article and approved the submitted version.
